# Non-covalent Interaction With SUMO Enhances the Activity of Human Cytomegalovirus Protein IE1

**DOI:** 10.3389/fcell.2021.662522

**Published:** 2021-05-13

**Authors:** Vasvi Tripathi, Kiran Sankar Chatterjee, Ranabir Das

**Affiliations:** National Centre for Biological Sciences, Tata Institute of Fundamental Research (TIFR), Bengaluru, India

**Keywords:** host-virus interaction, SUMO, NMR spectroscopy, cytomegalovirus, transactivation, Immediate early proteins (IE)

## Abstract

Viruses interact with the host cellular pathways to optimize cellular conditions for replication. The Human Cytomegalovirus (HCMV) Immediate-Early protein 1 (IE1) is the first viral protein to express during infection. It is a multifunctional and conditionally essential protein for HCMV infection. SUMO signaling regulates several cellular pathways that are also targets of IE1. Consequently, IE1 exploits SUMO signaling to regulate these pathways. The covalent interaction of IE1 and SUMO (IE1-SUMOylation) is well studied. However, the non-covalent interactions between SUMO and IE1 are unknown. We report two SUMO-Interacting Motifs (SIMs) in IE1, one at the end of the core domain and another in the C-terminal domain. NMR titrations showed that IE1-SIMs bind to SUMO1 but not SUMO2. Two critical functions of IE1 are inhibition of SUMOylation of Promyelocytic leukemia protein (PML) and transactivation of viral promoters. Although the non-covalent interaction of IE1 and SUMO is not involved in the inhibition of PML SUMOylation, it contributes to the transactivation activity. The transactivation activity of IE1 was previously correlated to its ability to inhibit PML SUMOylation. Our results suggest that transactivation and inhibition of PML SUMOylation are independent activities of IE1.

## Introduction

During the lytic cycle of herpesviruses infection, the Immediate Early (IE) genes are expressed first, followed by early and late viral genes. IE genes are multifunctional regulatory proteins that optimize the host cell environment for viral propagation. IE gene products help evade host antiviral responses, modulate the host cell cycle and transcribe viral genes. For Human Cytomegalovirus (HCMV), the two predominant IE proteins are IE1 and IE2, which are produced via alternative splicing of the mRNA from Major Immediate Early Promoter (MIEP) (Thomsen et al., 1984). IE1 is the first protein to express during HCMV infection and essential for a productive infection.

IE1 transactivates the host promoters and MIEP (Cherrington and Mocarski, 1989; Hayhurst et al., 1995). IE1 also enhances the transactivation activity of IE2 (Adamson and Nevels, 2020). IE1 can transactivate promoters by a variety of mechanisms. It enhances the expression of various cell-cycle proteins like DNA polymerase α, dihydrofolate reductase, or thymidine kinase by inhibiting transcriptional repressor p107 (Poma et al., 1996). IE1 inhibits chromatin-modifying enzymes like HDAC, ATRX, and DAXX to regulate transcription at the epigenetic level (Nevels et al., 2004; Adamson and Nevels, 2020). Additionally, IE1 tethers to the mitotic chromosome via its C-terminal chromatin tethering domain and alters higher-order chromatin structures (Zalckvar et al., 2013; Mucke et al., 2014; Fang et al., 2016). IE1 interacts with transcription factors like TAFII 130, SP1, CTF1, and E2F to transactivate the corresponding promoters (Margolis et al., 1995; Lukac et al., 1997; Yurochko et al., 1997).

Additionally, IE1 inactivates the host immune response (Knoblach et al., 2011; Wu et al., 2018). The phase-separated PML nuclear bodies (PML-NBs) are involved in the innate antiviral immune response (Kim and Ahn, 2015). They encapsulate and heterochromatinize the viral genome to repress transcription. Various DNA viruses inhibit PML-NB to alleviate transcriptional repression. The Promyelocytic leukemia (PML) protein has SUMO-Interacting Motif (SIM) and is SUMOylated at multiple sites. PML-NBs utilize heterotypic multivalent SIM/SUMO interactions between SUMOylated PML and its SIMs to phase separate (Shen et al., 2006). IE1 inhibits *de novo* PML SUMOylation to disrupt the PML-NBs (Torres and Tang, 2014). IE1 binds to STAT2 and inhibits its DNA binding, reducing type-I interferon signaling and ISG expression (Krauss et al., 2009; Torres and Tang, 2014). It also activates DNA damage response in an ATM-dependent manner (Castillo et al., 2005). IE1 activates PI3K/Akt pathway to prevent apoptosis of the host cell (Yu and Alwine, 2002). IE1 also inhibits p53 transactivation, where direct binding between IE1 and p53 inhibits p53-DNA binding (Hwang et al., 2009). The mechanism used by IE1 to regulate diverse host pathways is a subject of intense research.

SUMO is an integral part of the cell cycle regulation, transcription, and immune response pathways targeted by IE proteins. Hence, SUMO signaling is an apt target for the IE proteins to co-opt and regulate these pathways. The IE genes exploit SUMO signaling by either perturbing the SUMO machinery, SUMOylating host factors, or SUMOylating themselves. IE proteins like Ad E1B-55K, HCMV-IE2, EBV-Zta & RTA, HHV6-IE1B, KSHV-KbZIP, HPV-E1, and HPV-E2 are SUMOylated during infection. SUMOylation deficient mutants of these proteins are inactive and impede viral replication, highlighting the importance of SUMOylation (Lowrey et al., 2017). Non-covalent SIM/SUMO interactions regulate the localization and activity of several viral proteins like HCMV-IE2, KSHV-LANA, KSHV-L2, KSHV-KbZIP, and HSV1-ICP0 (Tripathi et al., 2019; Hembram et al., 2020; Mattoscio et al., 2020). Inhibiting the non-covalent interactions with SUMO depletes their activity and viral replication. Hence, studying the interaction between viral proteins and the SUMO is critical to understand how viruses exploit the host SUMO signaling. While IE1 SUMOylation is implicated in negative regulation of IE1-STAT2 binding (Spengler et al., 2002; Huh et al., 2008; Reuter et al., 2017), non-covalent interactions between IE1 and SUMO are unknown.

Here, we reveal that IE1 includes bonafide SIMs at the end of the core domain and C-terminal domains. Binding studies by NMR report that both the SIMs bind SUMO1 but not SUMO2, suggesting paralog specificity. Unlike the other immediate-early protein IE2, IE1 SIM/SUMO non-covalent interaction does not regulate IE1 SUMOylation. Neither does it play a role in inhibiting PML SUMOylation. However, the IE1-SIM1/SUMO interaction enhances the transactivation of IE2 responsive promoters. IE1 possibly recruits SUMOylated transcription factor or co-activators to IE2 responsive promoter. It is believed that transactivation due to IE1 is correlated to its PML-NB disruption activity (Lee et al., 2004). In contrast, our results indicate that the transactivation and PML-NB disruption mechanisms of IE1 are independent. Altogether, these results reveal an intriguing non-covalent interaction between IE1 and SUMO that enhances IE1 transactivation activity.

## Materials and Methods

### Plasmid and Peptides

IE1 peptides were synthesized from Lifetein (Table 1). SUMO1-pQE80L and SUMO2- pET15b were obtained from Rama Koti, TIFR, Mumbai. pET28- SUMO1(C-His) and pET28-SUMO2(C-His) were a kind gift from Dr. Christopher Lima, Sloan Kettering Institute, New York. Senp2 processed C-His-SUMO1 and C-His-SUMO2 to produce mature SUMO1/2 for SUMOylation assays. pET11-AOS1/UBA2 was also gifted by Dr. Christopher Lima. pET28-UBC9 was procured from Addgene (25213). Mammalian expression vectors, pDEST-SG5 HA-IE2 and pUL54-Luc, were gifted by Dr. Jin-Hyun Ahn, Sungkyunkwan University School of Medicine. HCMV-IE1 gene was synthesized from GeneArt (Thermo Fisher) and was cloned in pEF6 with C-terminal Myc and His tag. pEF6-IE1 was used as the template for SDMs to generate _335_VISV_338_-AAAA and _416_VIVA_419_-AAAA Myc-IE1 mutants. IE1 fragment (aa325-460) was synthesized by GeneArt (Thermo Fisher), which was cloned in pET14b with an N-terminal His tag and CFP to express His-CFP-IE1 (325–460).

**TABLE 1:** The affinity between IE1-SIMs and SUMO.

**SIMs**	**Resid#**	**Sequence**	**Kd (μM)**	
			**SUMO1**	**SUMO2**
IE1-SIM1	335–338	RPLITKPEVISVMKRRIEE	133.6 (±23.2)	No binding
IE1-SIM2	416–419	PATIPLSSVIVAENSDQEE	136.4 (±32.0)	No binding

### Protein Purification

BL21 (DE3) cells were used to express and purify all the proteins. BL21 (DE3) cells were cultured either in ^15^NH_4_Cl-M9 medium for labeled proteins and in LB for unlabeled proteins. Cells were cultured at 37°C to 0.8 OD_600_ and induced with 0.5 mM IPTG for 4–5 h. SUMO1-pQE80L and SUMO2-pET15b were used to express labeled N-terminal (His)_6_-tagged SUMO1/2 for titration experiments. Cells expressing ^15^N SUMO1/2 were lysed by sonication in a buffer containing 50 mM Na_2_HPO_4_ pH 8.0, 20 mM imidazole, and 300 mM NaCl. The lysate was clarified by centrifugation, and the supernatant was used to purify SUMO1/2 with Ni-NTA purification protocols. After Ni-NTA elution, fractions containing SUMO1/2 were concentrated and were further processed through a gel-filtration column (Superdex 75) in PBS buffer.

For SUMOylation assays, the mature forms of SUMO1 and SUMO2 were obtained by processing C-terminal (His)_6_-tagged SUMO1 or SUMO2 with SENP2. After Ni-NTA purification, fractions containing SUMO1/2 were incubated with purified His-ΔN364-SENP2 (1:1,000 molar ratio) at room temperature till complete digestion of C terminal extension. SENP2 and unprocessed SUMO were removed by passing the reaction mixture through Ni-NTA beads, and flow-through containing mature SUMO was collected and further purified with Superdex-75.

For *in vitro* SUMOylation assays, E1 (UBA2/AOS1) and E2 (UBC9) were purified with Ni-NTA affinity purification followed by Gel filtration as discussed in Yunus and Lima (2009). (His)_6_-CFP-IE1 (325–460) was purified using Ni-NTA affinity purification followed by SD75. CFP was used to probe IE1 in SDS PAGE gels. Lysis/wash buffer composition was 50 mM Tris pH 8, 350 mM NaCl, 1 mM PMSF, 1 mM beta-mercaptoethanol, 20 mM imidazole. Elution buffer contained 25 mM Tris pH8, 150 mM NaCl, 1 mM beta-mercaptoethanol, 250 mM imidazole and gel filtration buffer contained 20 mM Tris pH8, 50 mM NaCl, 1 mM beta-mercaptoethanol.

### Chemical Shift Perturbation

The ^15^N-HSQC spectra of SUMO1 and SUMO2 were recorded at 298K on an 800 MHz Bruker NMR Spectrometer. The spectra were processed with NMRpipe (Delaglio et al., 1995) and analyzed with Sparky (Kneller and Kuntz, 1993). Apo SUMO1 and SUMO2 were titrated with IE1-SIM peptides up to 1:4 (SUMO:SIM) ratio. On titration with the ligand, chemical shift perturbations were calculated and plotted against the residue number. 300 μM SUMO1/2 samples were prepared in PBS buffer, with 5 mM DTT and 10% D_2_O. For NMR titration experiments, IE1-SIM peptides were titrated into ∼0.3 mM ^15^N-SUMO1 or ^15^N-SUMO2. The titration data was fit in 1:1 protein: ligand model using the equation CSP_*obs*_ = CSP_*max*_ {([P]_*t*_ + [L]_*t*_ + K_*d*_) – [([P]_*t*_ + [L]_*t*_ + K_*d*_)^2^–4[P]_*t*_[L]_*t*_]^1/2^}/2[P]_*t*_, where [P]_*t*_ and [L]_*t*_ are total concentrations of protein and ligand at any titration point.

The mutant IE1 SIM peptides were purchased from Galore Tx Pharmaceuticals Private limited as lyophilized powders. The peptides were weighed in an analytical balance and subsequently dissolved in PBS. Mutant IE1-SIMs and 15N-wt-SUMO1 titration experiments were carried out in an 800 MHz Bruker Avance III HD spectrometer at 298K in PBS.

### SUMOylation Assay

For *in vitro* SUMOylation assays, 5 μM CFP-IE1 and 5 μM SUMO1/2 were incubated with 1 μM E1 and 2.5 μM E2. The reaction was started by adding ATP. Reaction buffer contains 20 mM HEPES pH 7.5, 50 mM NaCl, 5 mM MgCl_2_, 0.1% Tween 20. The reaction was stopped at the desired time points by adding reducing 5× SDS loading dye. The sample was never heated to keep CFP fluorescence intact. The reaction was analyzed either on a 12% SDS PAGE. Gels were imaged for CFP fluorescent signal.

For *in vivo* SUMOylation assays, HeLa or HEK-293T cells were cultures in a 12-well plate until 80% confluency. The cells were transfected with either 1 μg myc-IE1 (wt/mutants) or 500 ng SUMO1 and 500 ng myc-IE1 (wt/mutants). Cells were lysed 36–40 h post transcription in 2× SDS loading dye. Lysates were fractionated on 8% SDS PAGE gel and blotted with an anti-Myc antibody.

### Cell Culture and Transfection

HeLa and HEK-293T cells were cultured in DMEM with 10% FBS. Transformations were done with Fugene HD (Promega). For transactivation assays, cells were seeded into a 24 well plate and were cultured to 70–80% confluency. Cells were transfected at 70–80% confluency with 100 ng pUL54-Luc, 2 ng pTK-renilla (transfection control), 100 ng wt-IE2, and wt/mutant 100 ng Myc-IE1. Luciferase assay was performed 40–48 h post-transfection by Dual-Glo-luciferase kit (Promega). For PML degradation experiments, cells were transfected with 200 ng Flag-PMLIV and 300 ng Myc-IE1 in 6 well plates. Cells were lysed 40–48 post-transfection with 2×SDS loading dye. Lysates were fractionated on 8% SDS PAGE and blotted with Flag antibody to probe PML.

For the Microscopy experiments, HeLa cells were seeded in an 8-chamber coverslip plate (Eppendorf-0030742036). At 50% confluency, cells were transfected with 150 ng Flag-PML and 150 ng wt or mSIM1/2 Myc-IE1. Cells were cross-linked with 2.5% PFA, 36–40 h post-transfection. After washing with PBS, cells were blocked in 10%FBS + 0.5% TritonX100 in 1×PBS. Cells were incubated with Rabbit-anti-flag and mouse-anti-Myc primary antibody overnight at 4°C. After O/N incubation with primary antibody, cells were washed thoroughly with PBS and incubated for 1 h with Dapi anti-rabbit Alexa 488 and anti-mouse Alexa 562 secondary antibody. The samples were imaged in a Zeiss-apotome microscope.

## Results

### IE1-SIMs Bind to SUMO1

**FIGURE 1:**
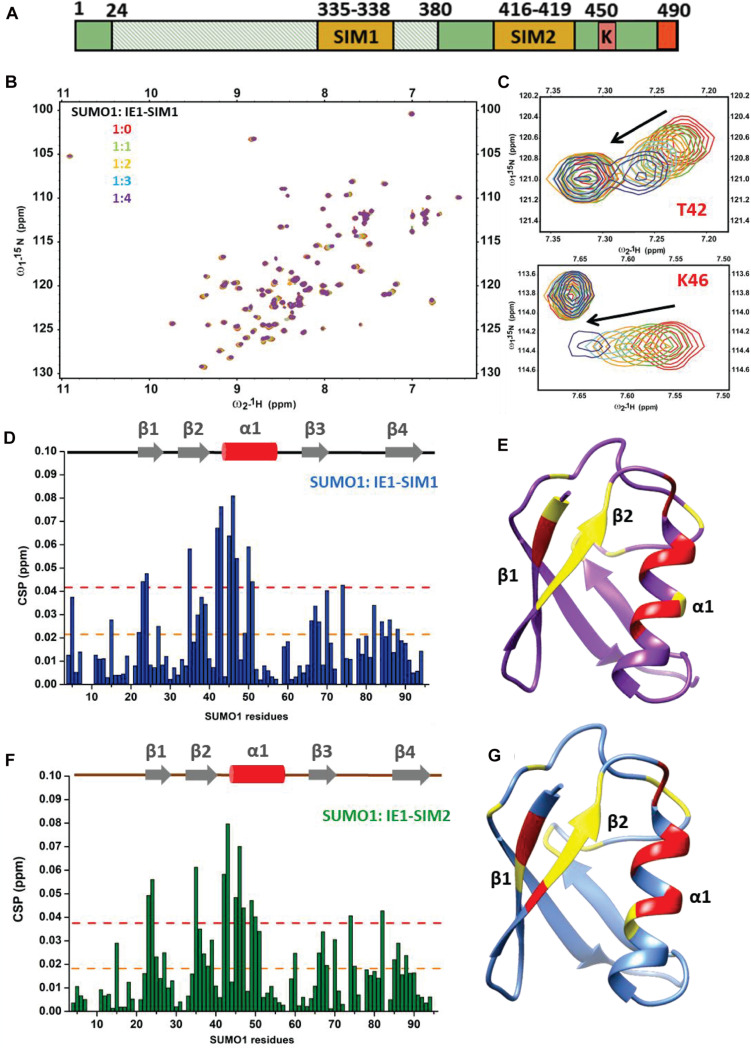
Interactions between IE1-SIM1 and SUMO1. **(A)** Representation of functional domains and putative SIMs of IE1. The shaded area from aa 24–380 is the core domain of IE1, responsible for inhibiting PML SUMOylation and its transactivation activity. C terminal domain of IE1 contains the STAT2 binding region, SUMOylation site, and chromatin tethering domain (in red) **(B)** Overlay of the ^1^H, ^15^N-edited HSQC spectra of free ^15^N-SUMO1 (red) with different stoichiometric ratios of IE1-SIM1. **(C)** Resonances of two residues of SUMO1 (T42 and K46) are expanded to show a shift of resonances during titration. **(D)** The CSPs upon binding to IE1-SIM1 are plotted against the residues of SUMO1. The CSPs are calculated as [(δN_*bound*_ – δN_*free*_)^2^/25 + (δH_*bound*_ – δH_*free*_)^2^]^1/2^, where δN_*bound*_ and δN_*free*_ are ^15^N chemical shifts of the amide resonance in bound and free form, respectively. Similarly, δH_*bound*_ and δH_*free*_ are ^1^H chemical shifts of the amide resonance in bound and free form. **(E)** The significant CSPs of IE1-SIM1 and SUMO1 binding are mapped onto the SUMO1 structure. **(F)** The CSPs upon binding to IE1-SIM2 are plotted against the residues of SUMO1. **(G)** The significant CSPs from **(F)** are mapped onto the SUMO1 structure. In **(D,F)**, the yellow dashed line indicates 1× standard deviation, and the red dashed line indicates 2× standard deviation. In **(E,F)**, the regions with CSPs above 1× standard deviation were colored yellow, and those with 2× standard deviation were colored red.

The HCMV-IE1 amino acid sequence was analyzed for putative SIMs. Bioinformatics analysis using JASSA (Beauclair et al., 2015) and manual inspection predicted two SIMs in IE1: SIM1 (aa 335–338) and SIM2 (aa 416–419) (Table 1 and Supplementary Figure 1). The binding of predicted IE1-SIMs to SUMO was studied by Nuclear Magnetic Resonance (NMR) spectroscopy. SIM/SUMO interaction is typically weak, and NMR can measure such interactions with high fidelity (Vaynberg and Qin, 2006). Ligand binding alters the chemical environment and the chemical shifts of the residues present at the interface. The changes in the chemical shifts are reflected in ^1^H, ^15^N-edited HSQC spectra. Peptides corresponding to the putative SIMs were designed (Table 1 and Figure 1A). The IE1-SIM1 was titrated into ^15^N isotope-labeled SUMO1, and a series of ^15^N-edited HSQC were recorded with increasing concentrations of IE1-SIM1 (Figure 1B). A few SUMO1 amide resonances shifted consistently with the increasing concentration of IE1-SIM1 (Figure 1C). Chemical Shift Perturbations (CSPs) measure the change in chemical shifts. The CSPs are calculated as [(δN_*bound*_ – δN_*free*_)^2^/25 + (δH_*bound*_ – δH_*free*_)^2^]^1/2^, where δN_*bound*_ and δN_*free*_ are ^15^N chemical shifts of the amide resonance in bound and free form, respectively. Similarly, δH_*bound*_ and δH_*free*_ are ^1^H chemical shifts of the amide resonance in bound and free form. The CSPs are plotted for SUMO1 in Figure 1D. Interestingly, maximum CSPs were observed between amino acids 35–50. These residues are part of beta-strand β2 and alpha-helix α1 of SUMO1 Figure 1E, the canonical interface for SIM/SUMO binding (Song et al., 2004). A hydrophobic shallow groove is present between β2 and α1 in SUMO, and the hydrophobic residues at the center of the SIMs bind to the groove (Hembram et al., 2020). Additionally, polar residues flanking with the central hydrophobic region interact to strengthen the binding. A fit of the CSPs against the ligand to protein concentration yields the dissociation constant (K_*d*_) of the complex. IE1-SIM1 binds to SUMO1 with a Kd of 134 (± 23) μM.

The NMR titration experiment was repeated for IE1-SIM2 and SUMO1 (Figures 1F,G and Supplementary Figure 2). IE1-SIM2 was titrated into ^15^N-labeled SUMO1, and ^1^H, ^15^N-edited HSQC spectra were collected with increasing concentration of IE1-SIM2 (Supplementary Figure 2A). Similar to IE1-SIM1, IE1-SIM2 also showed the highest CSPs in the region between β2 and α1 of SUMO1, suggesting that it is a bonafide SIM (Figures 1F,G). The fit of CSPs suggested that IE1-SIM2 binds SUMO1 with a dissociation constant of 136 (± 32) μM. IE1-SIM1 and IE1-SIM2 bind to SUMO1 with comparable affinities (Table 1). For control experiments, IE1-mSIM1 (VISV-AAAA) and IE1-mSIM2 (VIVA-AAAA) were designed and titrations were repeated with ^15^N-SUMO1. Unlike wt IE1 peptides, SUMO1 peaks were not significantly perturbed by IE1-mSIM1 or IE1-mSIM2 (Supplementary Figures 2C,D), indicating that SIM1 and SIM2 are bonafide SIMs.

SUMO2 is the other important paralog of SUMO1. The titration experiments of IE1-SIM1 and IE1-SIM2 were repeated with the ^15^N-labeled SUMO2 and monitored the HSQC spectra of SUMO2. Unlike SUMO1, SUMO2 peaks were not perturbed by SIMs (Supplementary Figure 3), indicating that IE1-SIMs do not interact with SUMO2. Altogether, IE1-SIM1 and IE1-SIM2 have paralog specificity, where the SIMs bind to SUMO1 but do not bind to SUMO2.

### IE1-SIMs Do Not Affect IE1 SUMOylation

IE1 and IE2 are the predominant immediate-early proteins expressed by HCMV. The IEs are SUMOylated (at K450 of IE1 and K175, K180 of IE2), and the SUMOylation modulates their function (Reuter et al., 2018). Interestingly, IE2 also includes a SIM in the N-terminal region (IE2-SIM), which increased its SUMOylation (Kim et al., 2010). SIM-enhanced *in-cis* SUMOylation is common in several host proteins like SP100, Daxx, PML, and TDG (Knipscheer et al., 2008; Kolesar et al., 2012). A putative function of the IE1-SIMs is to enhance IE1 SUMOylation. A fragment containing both the SIMs and SUMOylation site of IE1 (aa 325–460) was designed. The fragment was cloned with CFP at the N-terminus as a fluorescent tag. IE1 fused with similar tags have been utilized previously for experiments (Delmas et al., 2005; Vasou et al., 2018). Purified CFP-IE1 was used as the substrate for *in vitro* SUMOylation with SUMO1. After SUMOylation, the reaction was separated on SDS PAGE and was imaged for CFP. CFP-IE1 showed higher molecular weight bands after SUMOylation, but not CFP alone, indicating that IE1 was SUMOylated (Supplementary Figure 4).

**FIGURE 2:**
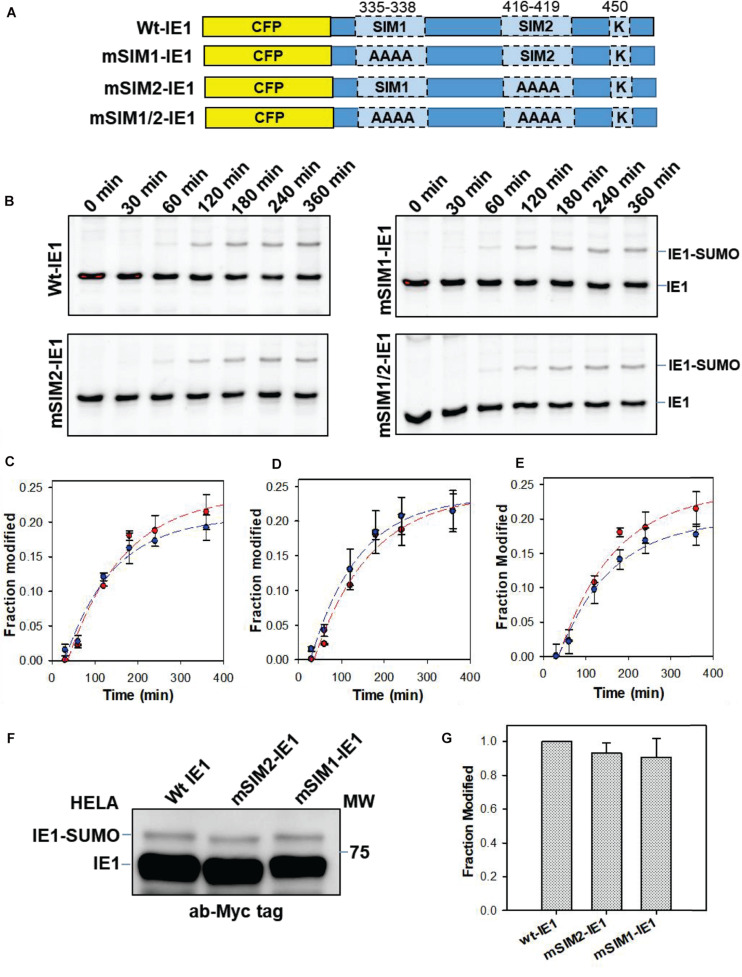
Effect of SIMs on SUMOylation of IE1. **(A)** The C-terminal fragment of IE1 (325–460, containing SIMs and SUMOylation site) was cloned with CFP. Schematic representations of SIM-mutants of IE1 are given. **(B)** IE1 (wt or mutants as mentioned) was SUMOylated *in vitro*. The reaction was stopped at given time points. The SUMOylation reaction was resolved on the SDS-PAGE gel and imaged for CFP. **(C–E)** The fraction of the IE1∼SUMO was quantified by ImageJ. Fraction modification is plotted against time. **(C)** represents wt and mSIM1-IE1, **(D)** represents wt and mSIM2-IE1, and **(E)** represents wt and mSIM1/2-IE1 (red is wt, and blue is the mutant). **(F)** HeLa cells were transfected with Myc-IE1 and SUMO1. Cells were lysed 48 h post-transfection were separated on the SDS page, and blotted with anti-Myc. The lower band indicates Myc-IE1, while the higher molecular size band indicates IE-SUMO. **(G)** IE1-SUMOylation in HeLa was repeated, and fraction SUMOylation was quantified.

IE1-SIMs were then substituted with alanines (_335_VISV_338_-AAAA, _416_VIVA_419_-AAAA), which are termed as mSIM1-IE1 and mSIM2-IE1, respectively (Figure 2A). *In vitro* SUMOylation assays were carried out with wt or mutant IE1 to assess the SIM’s relevance for IE1-SUMOylation. Interestingly, robust and rapid SUMOylation was observed for CFP-IE1 as well as the SIM mutants (Figure 2B). The extent of SUMOylation was quantified using ImageJ and was plotted against time to get the SUMOylation rate. The SUMOylation rate for wt or mSIM1 or mSIM2 was comparable (Figures 2C,D). A double mutant of SIMs was generated to test if one SIM’s absence is compensated by the other SIM. The double SIM mutant is termed mSIM1/2-IE1. SUMOylation of IE1 and mSIM1/2-IE1 was monitored over time. The rate of SUMOylation for wt CFP-IE1 was comparable to mSIM1/2-IE1. Substituting both the SIMs did not significantly affect the SUMOylation rate (Figure 2E), implying that IE-SIMs do not facilitate IE1 SUMOylation *in vitro*.

The importance of SIMs for IE1-SUMOylation was also tested *in vivo*. HEK-293T cells were transfected with wt/mutant Myc-IE1. However, a limited fraction of IE1 was SUMOylated with endogenous SUMO in cells (Supplementary Figure 5A). Thus, SUMO1 was transfected along with Myc-IE1 in HEK-293T cells (Supplementary Figure 5B). However, the SUMOylation of mSIM1-IE1 and mSIM2-IE1 was comparable to wt IE1. The experiment was repeated in HeLa cells, where the SUMOylation of IE1-SIM mutants was also comparable to wt IE1 (Figure 2F). As the SUMOylation was slightly greater in HeLa, we used HeLa-based IE1 blots to quantify IE1-SUMOylation. The fraction of IE1-SUMO was quantified for the wt and mSIM1/2 IE1 and plotted in Figure 2G. Taken together, the SIMs were dispensable for IE1 SUMOylation.

### IE1-SIMs Are Dispensable for PML-NB Dispersion

**FIGURE 3:**
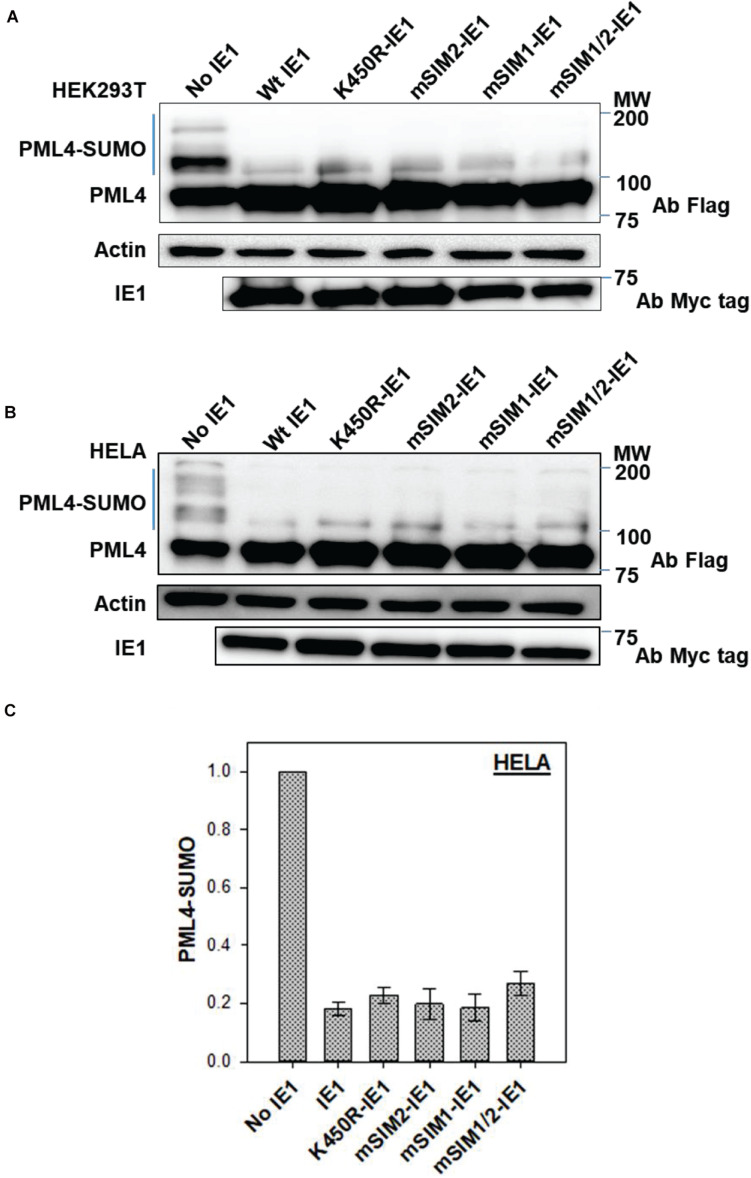
Inhibition of PML SUMOylation by IE1. **(A)** HEK- 293T cells were transfected with Flag-PML IV either without IE1 or with different IE1 mutants, as mentioned. Cells were lysed 40–48 h post-transfection. The lysate was resolved on 8% denaturing SDS PAGE and blotted with anti-Flag antibody. **(B)** HeLa cells were transfected and processed as mentioned for HEK 293T. **(C)** Blots from HeLa [as in **(B)**] were used to quantify PML SUMOylation in the presence of various mutants of IE1.

IE1 is the first viral protein expressed during HCMV infection and a key viral factor to evade host antiviral responses. PML nuclear bodies (PML-NB) are the hub for antiviral responses and are targeted by various DNA viruses, including HCMV (Tavalai et al., 2006). IE1 localizes to PML-NBs and disrupts it by inhibiting *de novo* PML SUMOylation (Schilling et al., 2017). Since PML-NB bodies are heavily SUMOylated, the relevance of IE1-SIMs for the inhibition of PML SUMOylation was investigated. HEK-293T cells were co-transfected with PML and IE1. In the absence of IE1, PML was highly SUMOylated (Figure 3A, first lane). As expected, PML SUMOylation is inhibited in the presence of IE1 (Figure 3A, second lane). The experiment was repeated with SIM mutants mSIM1, mSIM2, and the double mutant mSIM1/2. All three SIM mutants showed loss of PML-SUMO, implying that the SIM mutants are as active as wt IE1 (Figure 3A).

The experiment was repeated in HeLa cells, where mSIM1-IE1, mSIM2-IE1, and mSIM1/2-IE1 showed loss of PML-SUMO similar to wt IE1 (Figure 3B). The experiment was repeated (*n* = 3), and the fraction of SUMOylated PML was calculated for wt and SIM mutants of IE1 (Figure 3C). Co-transfection of mSIM1, mSIM2 or mSIM1/2-IE1 with PML showed equivalent fraction PML SUMOylation as wt IE1. PML SUMOylation was reduced by ∼80% in the presence of IE1. However, the loss of PML SUMOylation was similar for all the mutants, suggesting that the IE1-SIMs are dispensable for inhibiting PML SUMOylation.

The effect of IE1-SIMs on PML dispersion was studied by microscopy experiments. HeLa cells were transiently transfected with PML and wt or mSIM1/2-IE1. While cells transfected only with PML (blue arrow) showed PML-NB in the nucleus, cells co-transfected with wt IE1 and PML (white arrow) showed dispersed PML throughout the nucleus (Supplementary Figure 6). Cells co-transfected with mSIM1/2-IE1 and PML indicated that mutant IE1 could disrupt PML-NBs similar to wt IE1. The results also suggested that the mutations have not perturbed IE1structure as inhibition of PML SUMOylation was intact in these mutants. Altogether, the non-covalent IE1/SUMO interaction does not impact the inhibition of PML SUMOylation.

### IE1-SIM1 Is Essential for Enhancing IE2 Activity

**FIGURE 4:**
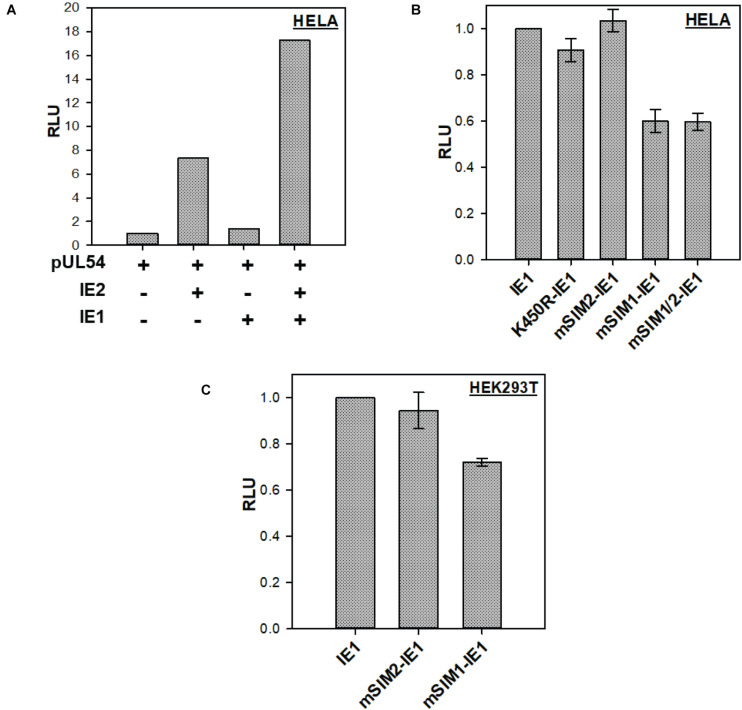
IE1-SIMs in IE1 enhanced IE2 transactivation activity: **(A)** Transactivation of pUL54-Luc by IE2 in the presence or the absence of IE1. **(B)** IE2 and pUL54-Luc were transfected in HeLa with wt or SIM mutants of IE1, as mentioned. Luciferase activity was tested 40–48 h post-transfection. The right panel shows an expression of IE2 and IE1 mutants (*n* = 3). **(C)** Similar to **(B)** but in HEK-293T.

IE2 is the major transactivator of the HCMV promoter. IE1 augments IE2 transactivation activity (Adamson and Nevels, 2020). The transactivation activity of IE2 is regulated by IE2-SIM (Kim et al., 2010). The role of IE1-SIMs in enhancing IE2 activity was tested. A typical luciferase reporter assay was used to analyze the IE1 activity. pUL54 promoter, one of the IE2 responsive promoters, was used in the luciferase reporter construct (pUL54-Luc). In HeLa cells, the basal activity of pUL54-Luc is low in the absence of IE2. As expected, co-transfection of IE2 with pUL54-Luc enhanced luciferase expression by several folds (Figure 4A, compare lane 1 and lane 2, Supplementary Figure 7A). IE1 alone failed to enhance pUL54-Luc luciferase expression (Figure 4A, lane 3). However, co-expression of IE1 and IE2 enhanced the luciferase expression from pUL54-Luc (Figure 4A, lane 4).

HeLa cells were further transfected with pUL54-Luc, wt-IE2, wt or SIM mutants of IE1. Luciferase expression was assayed 40–48 h post-transfection with the dual-luciferase assay, where pRL-TK (Renilla) was used as control. As seen in Figure 4B, SUMOylation deficient IE1 (K450R-IE1) showed similar activity as the wt IE1, implying that IE1 SUMOylation is not essential to enhance IE2 transactivation activity. Similarly, mSIM2-IE1 was as active as wt IE1, suggesting that IE1-SIM2 is dispensable for enhancing the IE2 activity. The observations can be explained as SIM2 and SUMOylation sites are present in the C-terminus of IE1, while IE1 transactivation activity resides at its N-terminal end. Interestingly, mSIM1-IE1 activity reduced to 40% in comparison to wt IE1 (Figure 4B). mSIM1/2 (where both the SIMs are mutated) showed activity comparable to mSIM1-IE1. The same experiment was repeated in HEK-293T cells, where mSIM1 had lower activity than wt IE1 (Figure 4C and Supplementary Figure 7B). Overall, IE1-SIM1 contributes to enhancing the IE2 transactivation activity.

## Discussion

IE1 performs several critical functions during the HCMV infection. IE1 disrupts the PML-NBs and inhibits STAT2 to evade antiviral responses. IE2 is the key transactivator of viral promoters. IE1 also enhances IE2 activity to promote viral gene expression. Interestingly, various viral proteins depend on SIM/SUMO non-covalent interaction for their activity. In this work, we identified two SIMs in IE1, SIM1 (aa 335–338) and SIM2 (aa 416–419), and studied the functional role of IE1/SUMO non-covalent interaction. The IE1-SIM1/SUMO and IE1-SIM2/SUMO non-covalent interaction is weak and similar to other SIM/SUMO interactions. Multiple ligand binding sites in a protein increase the local concentration of the ligand and has an additive effect, which increases the affinity of protein/ligand binding. The multiple SIMs in IE1 may provide the same effect to increase the net IE1/SUMO affinity. Alternately, phosphorylation increases the SIM/SUMO affinity, and SIM2 has multiple Serine residues, whose phosphorylation may modulate the interaction. The role of phosphorylation in the IE1/SUMO non-covalent interaction requires further investigation.

SUMOylation by different paralogs, i.e., either by SUMO-1 or SUMO-2/3, might lead to differential SIM/SUMO interaction and paralog-specific functional consequences. For instance, RanBP2-SIM has a higher affinity for SUMO1 than SUMO2/3, and thus, nuclear pores are SUMO1 (not SUMO2) enriched (Zhu et al., 2009). Alternately, CoREST1, which is a co-repressor, contains a SUMO2 specific SIM. Thus, CoREST1 is associated with transcriptional silencing of SUMO2-modified promoters (Ouyang et al., 2009). Interestingly, both IE1-SIM1 and IE1-SIM2 interacted with SUMO1 but not SUMO2. The functional relevance of paralog specificity in IE1 is a subject of future research.

SUMO-Interacting Motif enhances SUMOylation of SP100, Daxx, PML, and TDG. Various viral proteins (like HCMV-IE2 and KSHV-KbZIP) also show SIM-enhanced SUMOylation. However, both the *in vitro* and cellular experiments demonstrated that IE1-SIMs do not facilitate IE1 SUMOylation. The C-terminal domain is responsible for STAT2 binding and inhibition, which downregulates the antiviral responses. IE1-SIM2 also overlaps with the STAT binding site, suggesting that the fraction of IE1 bound to SUMO via SIM2 cannot bind STAT and inhibit IFN response. Instead, the SUMO bound fraction may contribute to other IE1 functions. IE1 SUMOylation adversely affects STAT2 binding (Huh et al., 2008). Hence, IE1 SUMOylation could be tightly regulated during infection, and the absence of SIM-enhanced SUMOylation correlates with the tight regulation. In HCMV-IE2, the SIM and SUMOylation sites are within twenty amino acids in the sequence. High-resolution structural models suggest that the IE-SIM interacts with Ubc9∼SUMO conjugate, bringing the conjugate in the vicinity of the SUMOylation site lysine to enhance SUMOylation (Tripathi et al., 2019). In HCMV-IE1, the SUMOylation site (K450) resides in the C-terminal acidic domain (Sadanari et al., 2005). The IE1-SIM1 (aa 335–338) is quite far in sequence from K450, while IE1-SIM2 (aa 416–419) is closer to K450. However, I-TASSER modeling indicates a structured alpha-helix between IE1-SIM2 and K450, suggesting a considerably extended and rigid intermediate region (Supplementary Figure 8). The presence of SIM-enhanced SUMOylation in IE2 but not IE1 suggests that the distance between SIM and the SUMOylation site and the intermediate region’s flexibility could be critical parameters for SIM-enhanced SUMOylation.

The constituent factors of PML-NB (like PML, Daxx, and sp100) are heavily SUMOylated (Shen et al., 2006) and utilize multivalent intermolecular SIM/SUMO interactions to phase separate. As PML-NBs are SUMO dense, various viral proteins like HCMV-IE2 and HSV1-ICP0 associate with PML-NBs and SUMOylated PML by their SIMs. IE1 also interacts with PML and inhibits its SUMOylation (Schilling et al., 2017). Interestingly, IE1-SIMs do not play a role in inhibiting PML SUMOylation. Our results suggest that IE1/PML interaction could be through their coil-coil motifs and not the SIM/SUMO binding.

**FIGURE 5:**
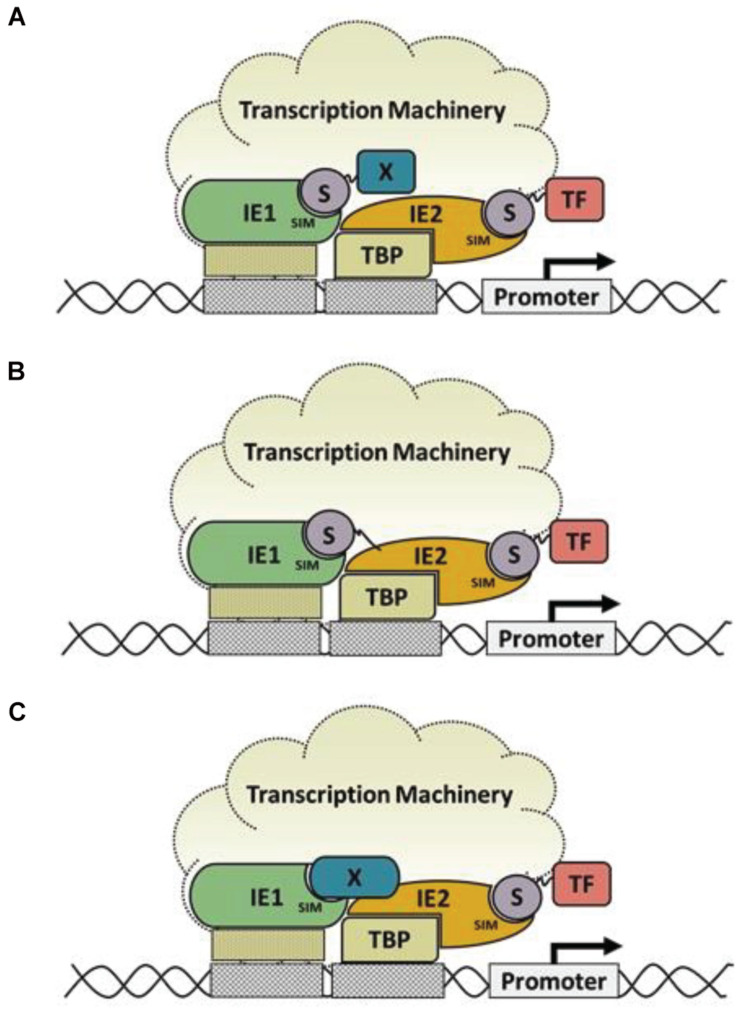
Models of IE1 enhanced IE2 transactivation activity: **(A)** A model of IE1-SIM1 enhanced transactivation activity, where IE1-SIM1 interacts with a SUMOylated transcription factor (X) to enhance IE2 transactivation. **(B)** An alternate model is where IE1-SIM1 interacts with IE2∼SUMO by non-covalent SIM/SUMO interaction. **(C)** Another possibility is that factor X interacts with IE1 on the surface, corresponding to IE1-SIM1 in SUMO independent manner.

IE1-SIM1 and IE2-SIM2 are present at the end of the core domain and the C-terminal acidic domain, respectively. The C-terminus of IE1 has no role in the transactivation of viral promoters, and our results suggest that the C-terminal IE1-SIM2 is dispensable for the same. The core domain has transactivation activity, and the IE1-SIM1 motif in this domain has a significant role in the activity. It would be interesting to study the SIM1 further by infection studies. The SIM/SUMO interactions have been shown to regulate transcription in many cases. For example, IE2-SIM recruits SUMOylated transcription factors (e.g., TAF12) for transactivation. Similarly, IE1-SIM1 may facilitate interaction with SUMOylated transcription factor or co-regulators (Figure 5A). Alternately IE1-SIM1 may enable interaction with IE2 via either SUMO-independent or SUMO-dependent mechanisms (Figures 5B,C). A direct interaction between IE1 and IE2 has not been reported yet, suggesting that the mechanism in Figure 5A is most likely.

Altogether, the current study uncovered and characterized non-covalent interactions between IE1 and SUMO. Two SIMs were identified in IE1, which interact with SUMO1, but not SUMO2. PML-NBs transcriptionally repress the viral genome. It is considered that IE1 transactivation activity is correlated to PML-NB disruption (Xu et al., 2001). Our results show that the SIMs are dispensable for PML-NB dispersion, but SIM1 contributes to the transactivation activity. Hence, the molecular mechanism underlying the two IE1 functions of PML-NB dispersion and transactivation are uncorrelated.

## Data Availability Statement

The original contributions presented in the study are included in the article/Supplementary Material, further inquiries can be directed to the corresponding author.

## Author Contributions

VT carried out experiments, analyzed the data, and wrote the manuscript. KC carried out experiments and analyzed the data. RD conceptualized and supervised the project, acquired funds, and wrote the manuscript. All authors contributed to the article and approved the submitted version.

## Conflict of Interest

The authors declare that the research was conducted in the absence of any commercial or financial relationships that could be construed as a potential conflict of interest.
