# Supplementary material for: Non-covalent Interaction With SUMO Enhances the Activity of Human Cytomegalovirus Protein IE1
Source: Front Cell Dev Biol. 2021 May 13;9:662522. doi: 10.3389/fcell.2021.662522 (PMC8155523; doi:10.3389/fcell.2021.662522)
Supplement: Supplementary file 1 [file Data_Sheet_1.PDF]

Non-covalent interactions with SUMO enhances the activity of Human Cytomegalovirus protein IE1

Vasvi Tripathi[1], Kiran Sankar Chatterjee[1], and Ranabir Das[1*]

[1]National Center for Biological Sciences, TIFR, Bangalore, India

Supplementary information

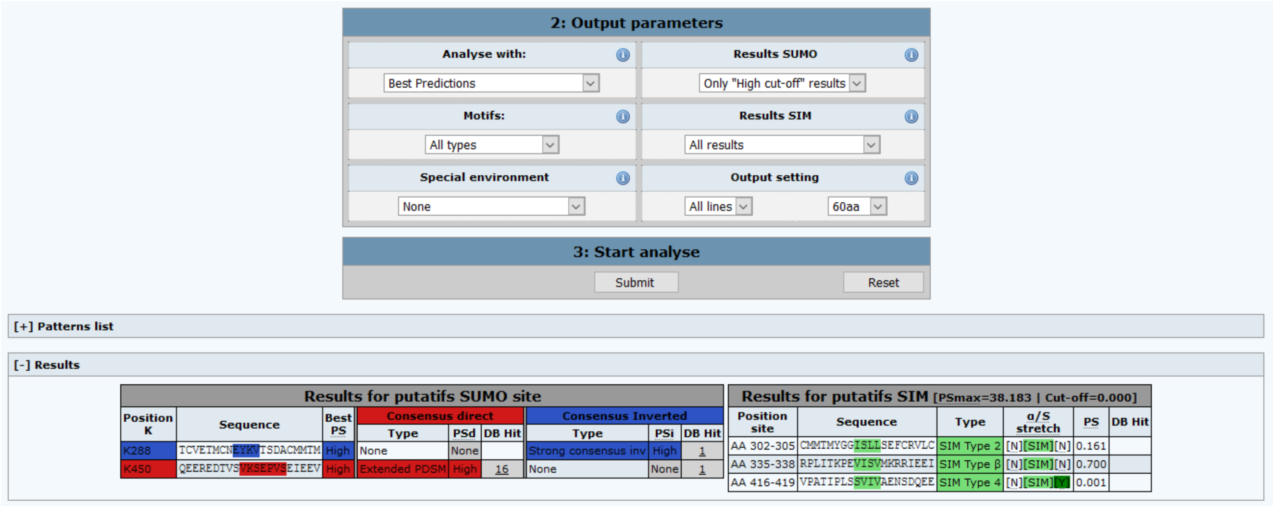

Figure S1: **Output of IE1 SUMOylation and SIM analysis by JASSA webserver.** Two SUMOylation sites and three putative SIMs were predicted by the server. Two of the SIMs were selected for further study by manual inspection of the sequence.

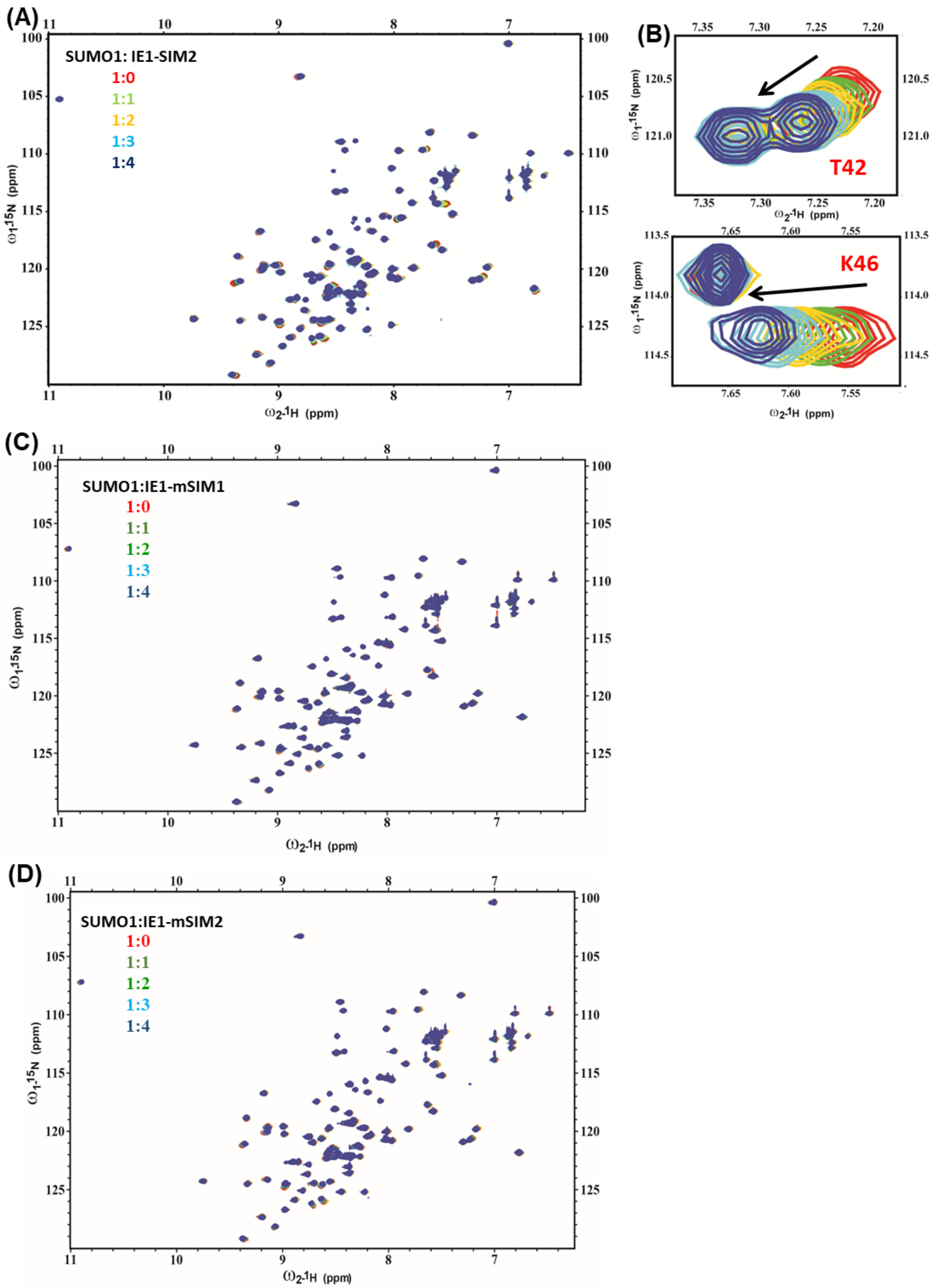

Figure S2: **Interactions between IE1-SIM2 and SUMO1** (A) Overlay of the ^{1}H, ^{15}N-edited HSQC spectra of free ^{15}N-SUMO1 (red) with different stoichiometric ratios of IE1-SIM2. (B) Resonances of two residues of SUMO1 (T42 and K46) are expanded to show a shift of resonances during titration. (C) Overlay of the ^{1}H, ^{15}N-edited HSQC spectra of free ^{15}N-SUMO1 (red) with different stoichiometric ratios of IE1-mSIM1. (D) Overlay of the ^{1}H, ^{15}N-edited HSQC spectra of free ^{15}N-SUMO1 (red) with different stoichiometric ratios of IE1-mSIM2.

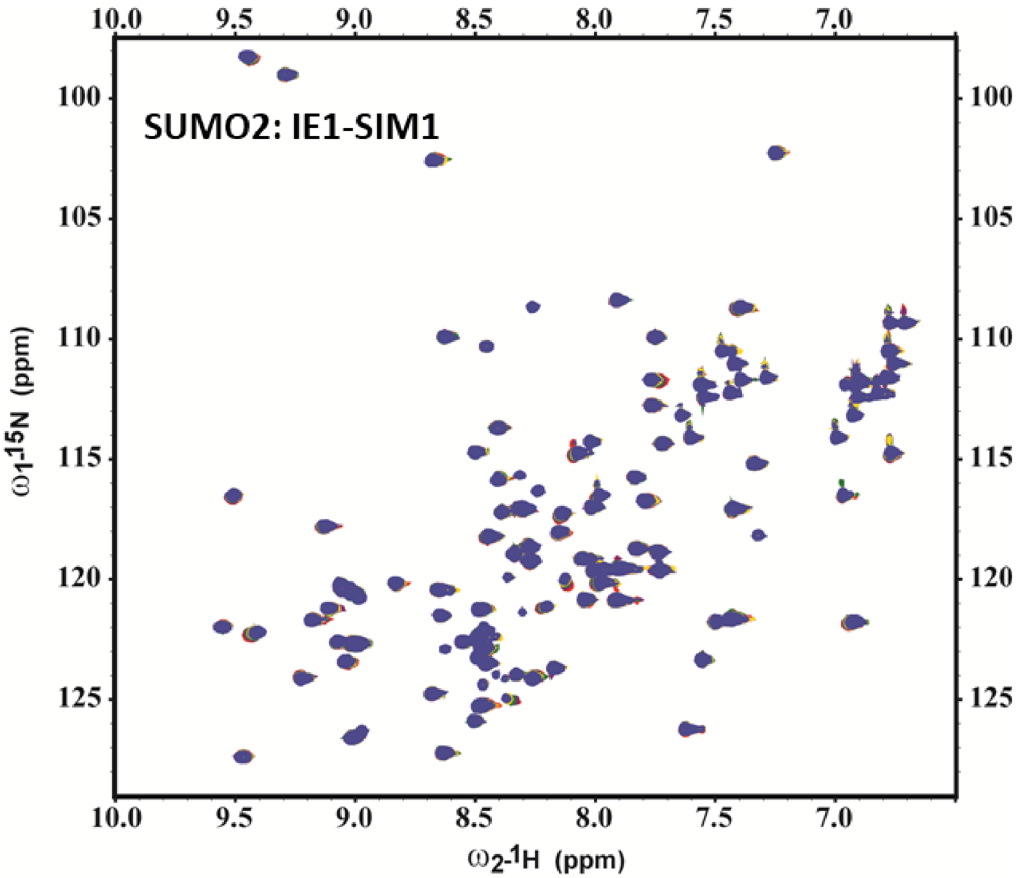

Figure S3: Interactions between IE1-SIM1 and SUMO2. An overlay of the ^{15}N-edited HSQC spectra of free ^{15}N-SUMO2 with different stoichiometric ratios of IE1-SIM1 has been plotted.

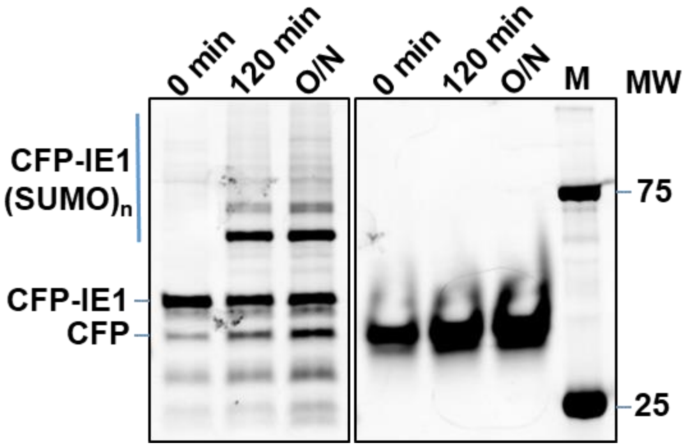

Figure S4: In-vitro SUMOylation of CFP-IE1 or CFP. CFP-IE1 or CFP was SUMOylated in-vitro. The reaction was stopped at given time points. The SUMOylation reaction was resolved on the SDS-PAGE gel and imaged for CFP. CFP was SUMOylated as a negative control.

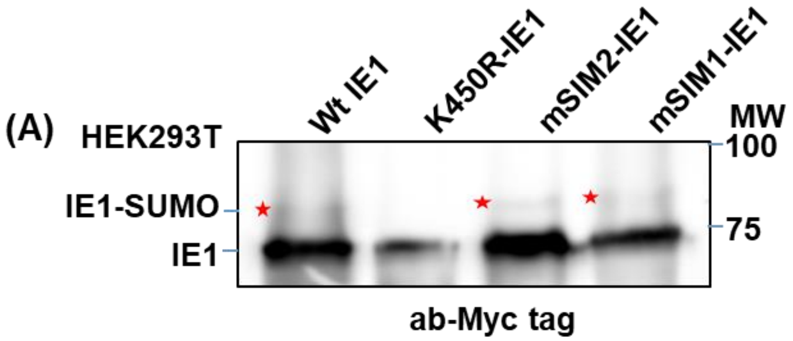

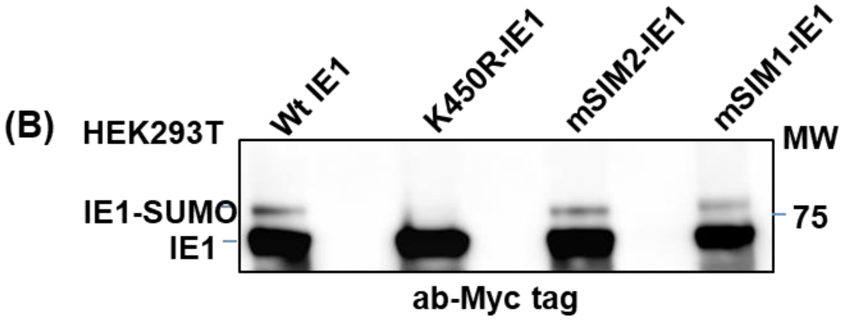

Figure S5: IE1 SUMOylation in HEK293T. (A) The SUMOylation of wt-IE1 and SIM mutated IE1 was observed in HEK293T cells. Cells were transfected with wt or mutant Myc-IE1. (B) HEK293T cells were transfected with an equal amount of Myc-IE1 and SUMO1.

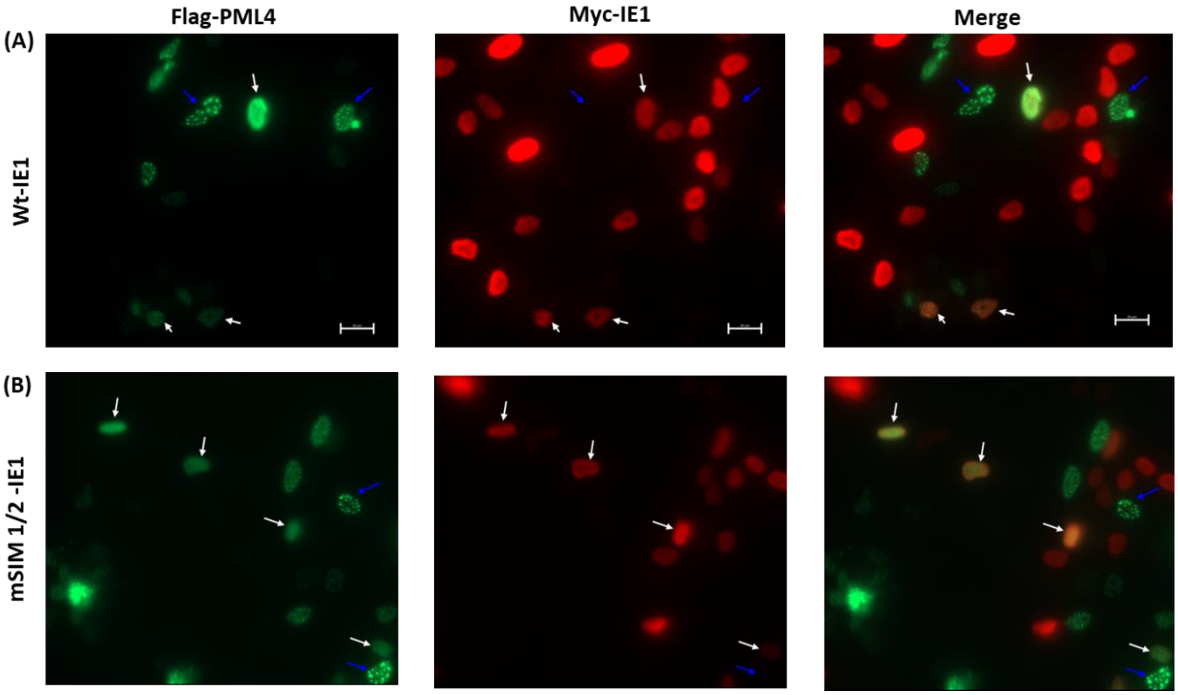

Figure S6: Figure S7: Dispersal of PML-NB in the presence of wt and mSIM1/2 -IE1. HeLa was transfected with (A) Flag-PMLIV with Myc-IE1 or (B) Flag-PML4 with Myc-mSIM1/2-IE1. The first panel shows Flag-PMLIV (represented in green), the second panel shows Myc-IE1 (represented in red), and the third panel is the merged image of the first two panels. In the panels, cells transfected only with Flag-PMLIV are marked by blue arrows, showing punctate PML distribution in the nucleus. While cells co-transfected with Flag-PMLIV and Myc-IE1 are marked with white arrows, showing dispersed PML throughout the nucleus. Scale denotes 20 µm.

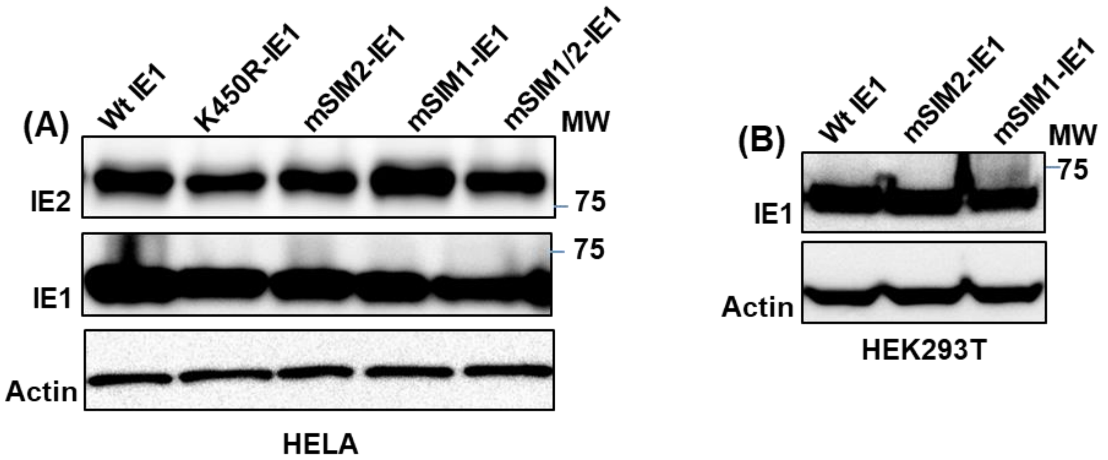

Figure S7: Expression profile of IE1 mutants in transactivation assay (Fig-6A, 6B).

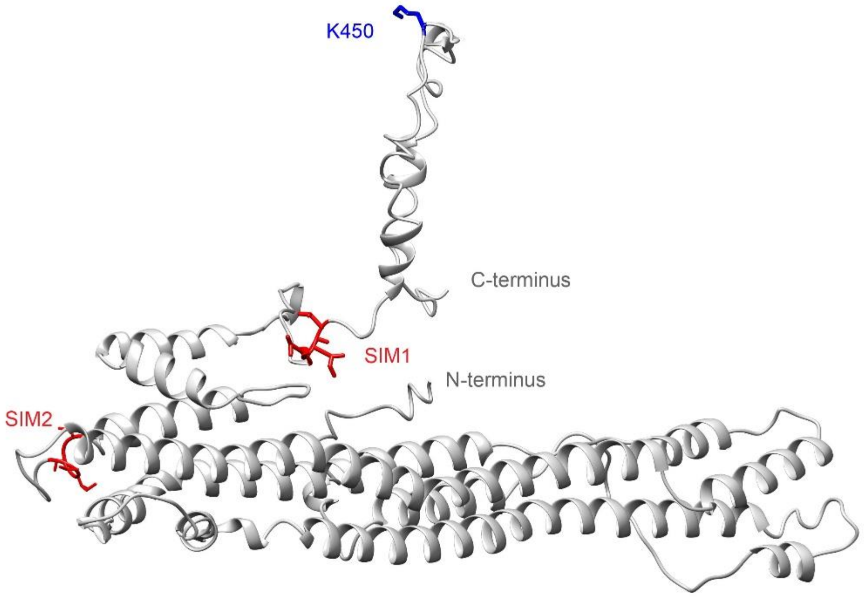

Figure S8: A structural model of IE1. A model of IE1 shows the position of SIM1 and SIM2 and the SUMOylation site K450. The atoms of SIMs and K450 are shown and colored in red and blue, respectively.
